# Resistance and germination of spores of *Bacillus* species lacking members of a spore integral inner membrane protein family and locations of these proteins in spores

**DOI:** 10.1128/jb.00217-25

**Published:** 2025-09-12

**Authors:** Shermeen Khan, James Wicander, George Korza, Rebecca Caldbeck, Ann E. Cowan, Graham Christie, Peter Setlow

**Affiliations:** 1 Department of Molecular Biology and Biophysics, UConn Health 705913, Farmington, Connecticut, USA; 2 Department of Chemical Engineering and Biotechnology, University of Cambridge 2152, Cambridge, United Kingdom; The Ohio State University, Columbus, Ohio, USA

**Keywords:** *Bacillus subtilis*, *Bacillus megaterium*, spores, spore resistance, spore germination, membrane fluidity

## Abstract

Spores of *Bacillus* species are dormant and resistant to heat and chemicals but “return to life” in germination, and cells of some species/strains can cause food spoilage or disease. Recently, two members of a family of five spore integral inner membrane (IM) small protein homologs were found to be important in *B. subtilis* spore resistance and germination. Among these IM proteins, YetF is the most abundant. In the current work, we show that loss of any of these five homologs decreased spore resistance to heat and chemicals, with greater decreases when multiple homologs were absent. In addition, *B. subtilis* spores’ loss of YetF and its homologs decreased the rates of spore germination, whereas loss of the second most abundant homolog, YrbG, increased germination rates. Surprisingly, *B. subtilis* spores lacking YetF and YrbG germinated spontaneously early in sporulation. Although this spontaneous germination did not involve normal germinant receptors or cortex peptidoglycan lytic enzymes, it was accelerated by overexpression of the IM channel for CaDPA release in germination. Loss of various homologs increased IM fluidity significantly, perhaps important in the effects of these proteins on spore resistance and germination. Notably, a functional YetF-GFP fusion was located in 5–7 IM spots in wild-type *B. subtilis* spores and in spores lacking the coat and outer membrane, although the function of these YetF spots is not clear. Similar spots were observed in *Bacillus megaterium* YetF-GFP spores, whereas *ydfS* null spores showed decreased wet heat resistance. Clearly, these novel proteins may have more surprises in store!

**IMPORTANCE:**

Spores of *Bacillota* are vectors for food spoilage and disease, and are hard to kill, as *B. subtilis* spores are killed only slowly by wet heat at 90°C. Multiple factors contribute to spores’ wet heat resistance, including low spore core water content and DNA-protective proteins. Recently, a group of spore-specific inner membrane (IM) proteins was identified as increasing IM rigidity and spore wet heat resistance. *B. subtilis* has five of these proteins, with multiple homologs in all *Bacillus* and *Clostridium* species. These proteins increase IM rigidity, which increases spore wet heat resistance and can either increase or decrease the rates of spore germination, with similar effects on *B. megaterium* spores. These proteins are thus a new factor important in spore properties.

## INTRODUCTION

Cells derived from the spores of some Firmicute species are major agents of food spoilage, food poisoning, and disease (1). A major reason for the effects of spores is their extreme resistance to many agents, including wet and dry heat, radiation at various wavelengths, and a host of chemicals (2). The most-studied spore resistance property is to wet heat, as spores of *Bacillus* species can withstand temperatures ~40°C higher than those tolerated by their vegetative cells (3). At least seven factors contribute to *B. subtilis* spore wet heat resistance (2), with some, such as spores’ accumulation of ~25% of core dry wt as the 1:1 chelate of Ca^2+^ with dipicolinic acid (CaDPA), identified ~70 years ago (4), and the seventh factor identified recently as two groups of homologous *B. subtilis* spore inner membrane (IM) proteins. Among these IM proteins, the loss of several homologous integral IM proteins was found to have large effects on spore wet heat resistance (5), whereas the four homologous peripheral IM lipoproteins had smaller effects (6). These IM proteins are made only in the developing spore late in sporulation and have significant effects not only on spore resistance but also on spore germination (5–7).

Germination of *B. subtilis* spores is usually triggered by germinants such as specific amino acids and sugars, which activate germinant receptors (GRs) in spores’ IM (8). GR activation then results in the opening of a spore IM SpoVA protein channel, leading to the rapid release of all core CaDPA. This CaDPA release then triggers the degradation of the spores’ large peptidoglycan cortex located between the germ cell wall outside the IM and the spore coat, by either of two redundant cortex-lytic enzymes (CLEs) CwlJ and SleB, and this leads to completion of spore germination.

The current work examines the effects of loss of one or more of the integral IM protein homologs noted above on the properties of spores of *B. subtilis* and *B. megaterium* including (i) resistance to wet heat, formaldehyde (HCHO), and hydrogen peroxide (H_2_O_2_); (ii) germination with different germinants as well as spontaneous germination; (iii) spore IM fluidity and permeability, and (iv) the specific location of two homologs of these proteins in spores’ IM. The findings in this work provide new insights into the features of dormant spores that are essential for their resistance and ability to return to life in germination and open a number of avenues for further investigation.

## MATERIALS AND METHODS

### Strain construction

The *B. subtilis* strains used in this work are isogenic with PS832, a laboratory 168 strain, and are listed in Table S1. Most mutant strains were constructed by transformation with genomic DNA from strains obtained from the *Bacillus* Genetic Stock Center, in which coding genes are replaced with an antibiotic resistance marker; in some cases, the antibiotic resistance genes were removed by the action of the Cre recombinase (9). In other cases, DNA from strains generated previously in the Setlow lab (5, 10, 11) was used for transformation. The genomic DNA from strains missing three or more homologs was also sequenced to confirm the genotypes of these strains. *B. subtilis* wild-type (wt) and Δ*yetF* strains expressing YetF fused to GFP and under the control of the wt *yetF* promoter were generated by cloning a PCR amplicon encoding 200 bp of upstream sequence from the *yetF* start codon plus the *yetF* ORF fused in frame at the 3’ end with the superfolder *gfp* ORF into plasmid pDG1662 AmyE integration vector. The resultant vector was introduced into competent wt and Δ*yetF B. subtilis* strains that were subsequently verified by PCR and sequencing.

The *B. megaterium* Δ*ydfS* strain was prepared by allelic exchange, introducing a truncated version of the gene additionally interrupted with a kanamycin resistance cassette at the *ydfS* locus (BMQ_1954) using the PEG-mediated transformation protocol described previously (12). The pUCTV2 Δ*ydfS*::Km plasmid was prepared by Klenow Assembly, where PCR amplicons encompassing 500 bp of the 5’ and 3’ ends of the *ydfS* ORF were positioned on either side of a kanamycin resistance cassette amplified from plasmid pDG792 and cloned within linearized temperature-sensitive pUCTV2. The *B. megaterium* Δ*ydfS* strain, which was isogenic with the wt QM B1551 strain, was verified by PCR and sequencing. As is often the case, the mutagenesis procedure led to the loss of the native 165 kb pBM700 plasmid, which carries *gerU* and associated *gerVB* germinant receptor genes, and BMQ_pBM70026, which encodes a *YetF* homolog. Loss of g*erU* was circumvented in the Δ*ydfS* strain by introducing a copy of the hybrid *gerUV* operon on the low-copy episomal pHT315 plasmid. This plasmid was also used to complement the Δ*ydfS* strain using a *ydfS-gfp* construct (prepared by Klenow Assembly and which included a GGGGS flexible linker between *yetF* and superfolder *gfp* ORFs), which was preceded by 200 bp of upstream sequence, that is, placing the constructs under control of the native promoter sequence. A plasmid encoding *B. megaterium yetF-gfp* was prepared in a similar manner (YetF is encoded at BMQ_2888) and introduced to wt cells by PEG-mediated transformation.

### Spore preparation and purification, and spontaneous spore germination

*B. subtilis* strains were sporulated on 2× SG medium agar plates at 37°C as described previously (13, 14). Routinely, after ~3 days when spore release from sporangia was largely complete, the spores were scraped from plates into cold water, and they were purified by repeated centrifugation, water washes, and intermittent sonication, as described (15). The final purification step was centrifugation through a high-density solution of 50% Histodenz, in which the dormant spores pellet and germinated spores and debris float. The pelleted spores were then washed with cold water to remove Histodenz and stored at 4°C, protected from light at an optical density (OD_600_) ranging from 10–30. The final spore preparations were also examined intermittently by phase contrast microscopy to ensure that spores remained dormant. This was the case for almost all spore preparations except for some strains lacking multiple IM homologs, which, although initially giving phase bright spores in sporangia, exhibited large percentages of germination soon after mother cell lysis (see Results).

In one experiment, the spores were left on plates at 37°C for 48 h, and small samples were intermittently scraped from small areas of plates and examined by phase contrast microscopy to determine the percentages of dormant (phase bright) and germinated (phase dark) spores; the samples were also stained with the BacLight reagent to identify spores that had germinated and were alive and staining green or were dead and staining red (16). In a 6-day incubation of sporulation plates, the spores were scraped from equal small areas of plates at various times, suspended in 2 mL of cold water, and centrifuged, and the supernatant was saved; 2 mL of water was added to the pellet, which was suspended, boiled for 30 min to release CaDPA, cooled, and centrifuged, and again, the supernatant was saved. Aliquots (5, 10, and 25 µL) of both supernatants were added to a fluorescence cuvette to give 200 µL of 25 mM K-Hepes buffer (pH 7.4) and 50 µM TbCl_3_, and the Tb-DPA fluorescence was measured fluorometrically as described (5).

*B. megaterium* spores were prepared in supplemented nutrient broth (SNB) in a shaking (225 rpm) incubator for 72 h at 30°C and harvested and purified as described previously (12). Spontaneous germination of *B. megaterium* spores was assessed by sporulating wt and Δ*ydfS* strains in SNB as described above, and then reducing orbital agitation to 100 rpm after 72 hr prior to withdrawing samples for phase contrast microscopy and imaging over a 25-day period.

### Spore killing

To assess *B. subtilis* spore killing by wet heat, spores at an OD_600_ of ~1 (~10^8^ spores/mL) were incubated in sterile water at either 90°C or 93°C. At various times, 50 µL aliquots were added to 450 µL of cold sterile water, and the one-tenth dilutions were further diluted serially 10-fold up to 1/10^6^ in sterile cold water. Spore viability was determined by spotting 10 µL aliquots in duplicate in a grid on lysogeny broth (LB) medium plates (14), with appropriate antibiotics as required. After the applied liquid had dried on the plates, they were incubated overnight at 30°C and then at 37°C until no more colonies appeared, and colonies were counted. Spore killing by H_2_O_2_ and HCHO at 23°C was as described previously (17), and the aliquots were diluted one-tenth in solutions to inactivate the chemicals used, incubated for ~30 min, and then serially diluted one-tenth further in water, and the aliquots of multiple dilutions were applied in duplicate to LB plates, which were incubated, and colonies counted as described above for analysis of wet heat resistance.

Wet heat resistance of *B. megaterium* spores was assayed by incubating 100 µL aliquots of spores in sterile water (OD_600_ of 1; ~10^8^ spores/mL) at 75°C. Samples were taken after 30, 60, and 90 min, cooled, and diluted 10-fold with ice-cold sterile water, and then serially diluted in water before plating in triplicate on LB plates. Colonies were counted after overnight incubation at 30°C.

### Spore germination

Spores of *B. subtilis* strains were germinated with either the GR-dependent germinants L-valine or a mixture of L-asparagine, D-glucose, D-fructose, and KCl (AGFK), or the non-GR-dependent germinant dodecylamine, which directly opens the SpoVA IM CaDPA channel. Spore germination by L-valine, AGFK, and dodecylamine was measured by monitoring CaDPA release by DPA fluorescence with Tb^3+^ as described (18). The GR-dependent germination described above was preceded by a heat shock at 70°C for 30 min (L-valine) or 2 h (AGFK) (19) to synchronize germination, but is not needed for dodecylamine germination (8). Germination incubations of 200 µL were: (i) at 37°C with 10 mM L-valine in 200 µL of 25 mM K-Hepes buffer (pH 7.4) plus 50 µM TbCl_3_, with spores at an OD_600_ of 0.5, and (ii) at 37°C in 10 mM each L-asparagine, D-glucose, D-fructose, and KCl in 25 mM K-Hepes buffer (pH 7.4) with spores at an OD_600_ of 0.5, plus 50 µM TbCl_3_. Germination was begun by spore addition, and Tb-DPA fluorescence was measured in a fluorescence plate reader (18). For dodecylamine germination, spores at an OD_600_ of 1 were incubated at 45°C in 2 mL of 1 mM dodecylamine in 5% DMSO and 25 mM K-Hepes buffer (pH 7.4). At various times, 100 µL aliquots were added to 100 µL of 100 µM TbCl_3_ in 25 mM K-Hepes buffer (pH 7.4), and Tb-DPA fluorescence was measured. All germinations were carried out in duplicate, and the data were averaged.

*B. megaterium* spore germination was monitored by absorbance loss and DPA release assays using spores that had been heat-activated at 60°C for 10 min and subsequently cooled on ice. Absorbance assays were conducted using 96-well plates, adding 20 µL of spores at an OD_600_ of 4–180 µL aliquots of 5 mM Tris-HCl, pH 7.5, supplemented with 10 mM D-glucose, giving an initial OD_600_ of 0.4. Plates were sealed with adhesive film to prevent evaporative losses and incubated at 30°C in a Tecan Infinite-200 series shaking incubating plate reader fitted with a 600 nm photometric filter. Plates were subjected to 10 s orbital shaking after each absorbance measurement, which was collected every minute over a 2 h period. Germination assays for DPA release entailed the addition of 20 µL spores to black microtiter wells containing 180 µL of the glucose-Tris buffer mix noted above plus 50 µM TbCl_3_. DPA release was measured by monitoring fluorescence emission at 620 nm with excitation at 337 nm for 90 min at 30°C using a BMG Labtech CLARIOstar Microplate Reader operating in homogeneous time-resolved fluorescence mode with orbital shaking. Germination experiments were conducted with at least two independently prepared batches of spores, and where SD from mean values was <10%.

### Fluorescence redistribution after photobleaching (FRAP) analysis, and other measurements of IM fluidity and permeability

Developing *B. subtilis* spores were labeled with ~5 µM 2,4-di-ANEPPS dye in liquid sporulation, and spores were purified as described above. The purified labeled spores were subject to analysis by fluorescence redistribution after photobleaching (FRAP), essentially as described previously (20), except that diffusion coefficients and mobile fractions were estimated by comparing experimental results with simulated results using a 3D computational model of the FRAP experiment developed in Virtual Cell (VCell) software (21, 22). The VCell BioModel “Bacillus Spore Membrane new FRAP Analysis” under username “ACowan” can be accessed within the VCell software available at https://vcell.org. The VCell BioModel is modified from the BioModel described in (23) and accounts for bleaching during monitoring and unquenching after bleaching, both determined in control experiments, as well as diffusion during the redistribution after photobleaching.

As another measurement of spore IM fluidity, *B. subtilis* spores of the wt and PS4531 strains were labeled with 50 µg/mL Laurdan (6, 24) in the plates used for spore preparation, and the spores were prepared and purified as described above. Note that the Laurdan that is taken up remains in purified spores and in the IM (6, 24). Laurdan fluorescence was excited at 405 nm, and fluorescence emission at 440 and 490 nm was measured for 30–40 individual spores, including several germinated spores, and the ratios of the emission intensities at the two wavelengths were taken as a measure of IM fluidity (6, 24). The permeability of ^14^C-methylamine across the spore IM and into the dormant spore core was also measured as described previously (23).

### Fluorescence microscopy of spores carrying various fluorescent fusions

Fluorescence and brightfield images of *B. subtilis* spores carrying YetF-GFP were obtained using Zeiss Elyra 7 Super-resolution Structured Illumination Microscopy (SR-SIM). Intact spores (5 µL in water) were spotted onto 1% agarose-coated glass slides, followed by the addition of a coverslip. Fluorescence imaging was obtained by two-dimensional SIM with laser excitation at 488 nm (argon laser) and emission collected at 515 nm with a 63× objective (alpha Plan-Apochromat 63×/1.46 Oil). Brightfield images were obtained using transmitted light and filter set BP 570-620 + LP 655. Image processing and presentation were performed with Fiji software and Adobe Photoshop. *B. megaterium* spores were imaged using an Olympus BX53 microscope (Olympus Life Sciences, UK) fitted with a x100 oil immersion objective, phase contrast optics, and filter sets for acquisition of green fluorescence. The resultant images were processed using Fiji software (ImageJ).

## RESULTS

### *B. subtilis* spore resistance

**Fig 1:**
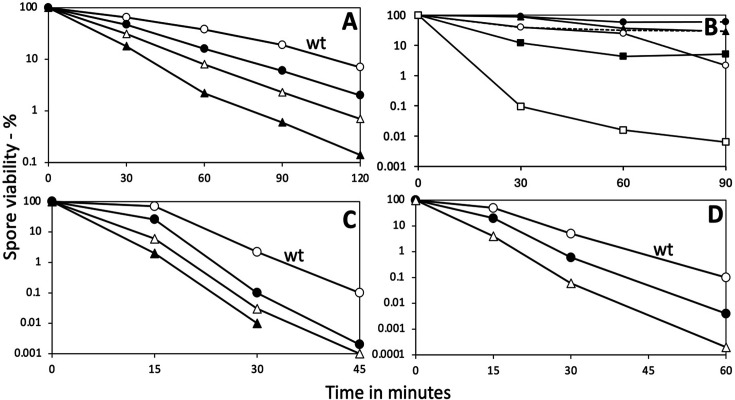
(A–D) Killing of *B. subtilis* spores (panels A, C, D) or *B. megaterium* spores (panel B) with and without the various YetF homologs by: (A) wet heat (90°C); (**B**) wet heat (75°C or 85°C); (**C**) H_2_O_2_; (**D**) HCHO as described in Methods; all values are averages of duplicate determinations that differed by <20%. Symbols denoting the homologs absent are: in (A) 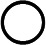 - none (PS832);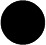 ˜ – *yrbG* (PS4517); 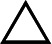 r – *ydfR yrbG* (PS4520); 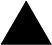 *P* – *ydfR ykjA yrbG* (PS4524); in (B) 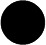 and 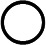
– none (QM B1551 wt at 75°C and 85°C, respectively); 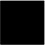 and 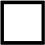 - *ydfS* at 75°C and 85°C respectively; 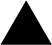 – *ydfS* with plasmid-borne *ydfS-gfp* at 75°C; dashed line – *ydfS* with plasmid-borne *gerUV* at 75°C; in (C) 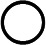 - none (PS832); 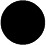 – *yrbG* (PS4517); 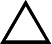 – *yrbG ydfR* (PS4520); 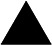 – *yrbG ykjA ydfR* (PS4524); and in (D) 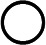 - none; 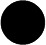 – *yrbG* (PS4517); and 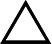 – *yrbG ykjA ydfR* (PS4524).

Previous work (5) showed that loss of either YdfS or YetF, two similar homologs of the *B. subtilis* family of five integral spore-specific IM proteins, resulted in decreases in spore resistance to wet heat, as well as to H_2_O_2_ and HCHO. Thus, it was of interest to examine the resistance properties of spores lacking genes encoding any one of the other three homologs or lacking multiple homologs (Fig. 1A, C, and D). Not surprisingly, loss of *yrbG*, *ydfR,* or *ykjA* alone or together resulted in decreases in spore wet heat resistance (Fig. 1A), with the triple mutant retaining only YdfR and YetF (strain PS4531) having the lowest heat resistance. We made the quintuple mutant lacking all five homologs, but this strain was very unstable and germinated rapidly after release from the sporangium, as did spores lacking both *yetF* and *yrbG*, and either with or without other homologs (see below). The cumulative effect of loss of multiple homologs of these proteins was also seen when resistance to H_2_O_2_ or HCHO was examined, with the triple mutant retaining both YetF and YdfS giving the lowest resistance to these two agents (Fig. 1C and D).

### *B. subtilis* spore germination

**Fig 2:**
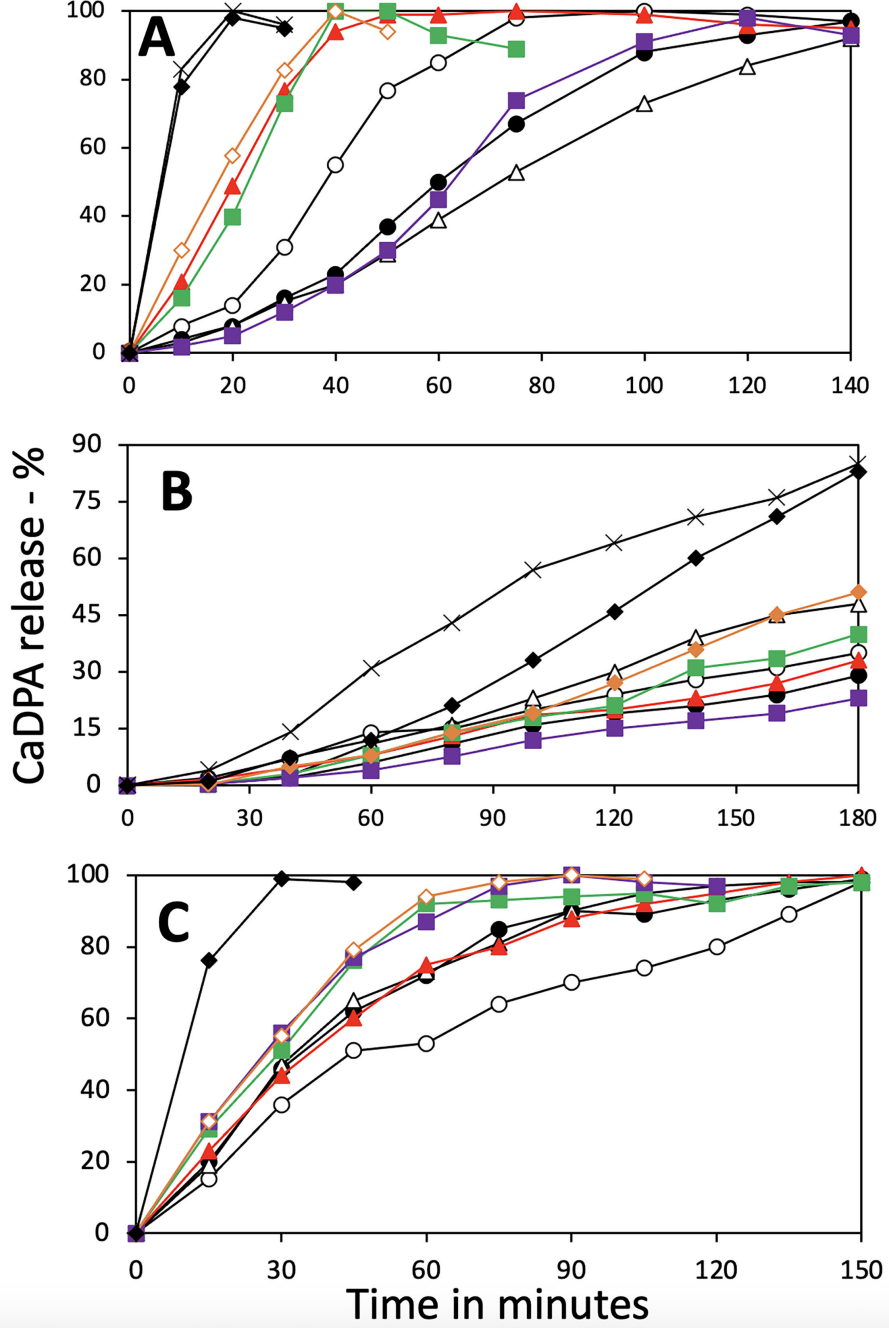
(A–C) Germination of *B. subtilis* spores with and without various YetF homologs with (A) L-valine; (**B**) AGFK; or (C) dodecylamine as described in Methods; all values are averages of duplicate determinations that differed by <15%. Symbols in (A), (**B**), and (C) denote the homologs absent: in (A) and (B) 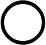 - none; 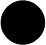 – *ydfS* (PS4483) (black); 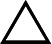
– *yetF* (PS4484); 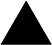 (red)– *yrbG* (PS4517); 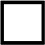 (green) - *ykjA* (PS4518); 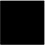 (purple)- *ydfR* (PS4519); 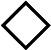 (orange)- *ydfR yrbG* (PS4520); 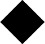 (black)- *ydfR yrbG ykjA* (PS4524); X – *yrbG ykjA ydfS* (PS4531); and in (C) 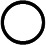 - none; 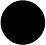 (black)– *ydfS*; 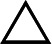 – *ykjA*; 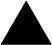 (red)– *ydfR*; 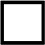
(green)- *yrbG*; (purple)- *yetF*;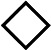 (orange)- *ydfR yrbG ykjA*; 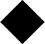 (black) *yrbG ykjA ydfS*.

Previous work (5) also showed that loss of either YetF or YdfS caused significant slowing of spore germination with the GR-dependent germinant L-valine, compared with wt spores. This was also seen in the current work, and loss of *ydfR* caused a similar slowing of germination (Fig. 2A). However, loss of either *yrbG* or *ykjA* resulted in more rapid L-valine germination than with wt spores, and this effect was even more pronounced in double and triple mutants. Effects similar to those on L-valine germination were also seen with AGFK germination, which was slower in some single mutants, but again, double and triple mutant spores generally germinated significantly faster with AGFK than wt spores (Fig. 2B). The effects of these mutations on dodecylamine germination were different, as spores of all mutants germinated more rapidly than wt spores with this GR-independent germinant (Fig. 2C), and spores of a triple mutant exhibited the most rapid dodecylamine germination.

**Fig 3:**
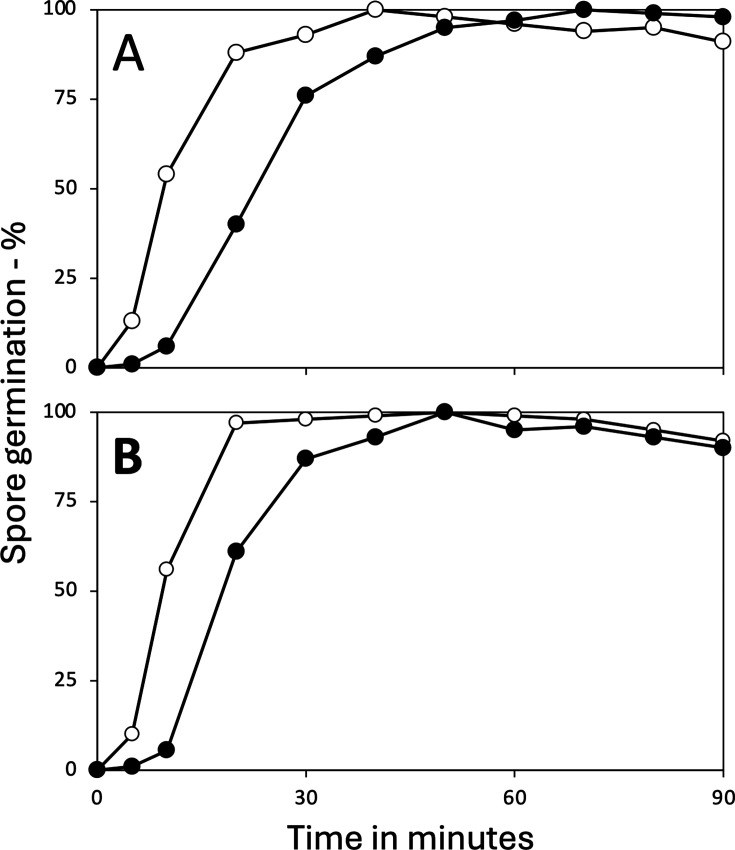
(A, B) Effects of antibiotic markers on L-valine germination of spores of *B. subtilis* strains lacking YetF homologs. Strains used are: (A) PS4531 and PS4601 that lack *yrbG*, *ydfS*, and *ykjA* genes, and either carry genes for resistance to Erm or Kan (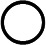) or lack these genes (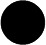), and (B) PS4524 and PS4602 that lack *yrbG*, *ydfR,* and *ykjA* genes and either carry the gene for resistance to Kan (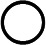) or lack this gene (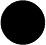). Purified spores of both strains were heat shocked, cooled, and duplicate samples germinated as described in Methods, and germination was monitored by measuring CaDPA release as described in Methods; all values shown are averages of duplicate determinations.

We also tested the effects of antibiotic markers on the germination of *B. subtilis* spores lacking three or four homologs (Fig. 3A and B), since recent work has shown that integrated exogenous DNA can have significant effects on spores’ proteomes (25) and thus presumably on spore properties. Indeed, the otherwise isogenic spores with one or two antibiotic marker(s) germinated more slowly when the antibiotic markers had been removed. However, the reason(s) behind this effect were not studied further, although this phenomenon needs to be kept in mind when working with spores of strains carrying antibiotic markers.

### Spontaneous germination of spores lacking both YetF and YrbG

**Fig 4:**
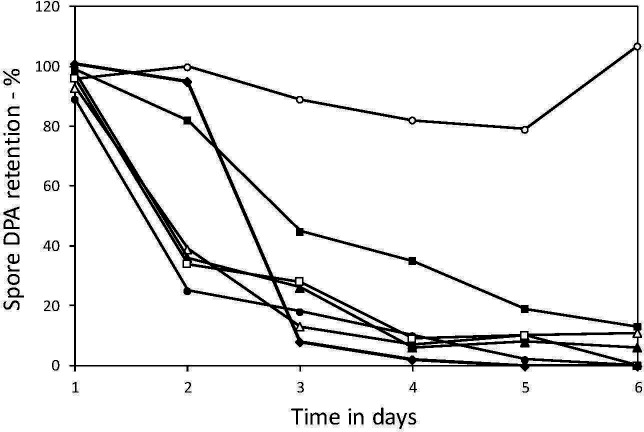
Spontaneous CaDPA release in sporulation by *B. subtilis* spores lacking YetF homologs and germination components. Sporulation on plates and monitoring DPA retention in spores scraped from plates at various times were as described in the methods. The strains used are isogenic with wild-type PS832, and symbols used are: 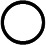 - wild-type; (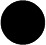) – PS4530 lacking all *yetF* family homologs except *ydfR*; 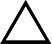 – PS4533 lacking *yetF* and *yrbG*; 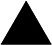 – PS4536, PS4530 also lacking *cwlJ*; - PS4541, PS4533 also lacking genes for all GRs (PS4501); 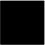 - PS4542, PS4530, also lacking *cwlJ* and *sleB*; and 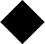
- PS4540, PS4530 lacking *sleB*. Note that spores of the mutant strain’s completion of germination as observed by phase contrast microscopy closely paralleled CaDPA release, except for PS4542 spores that only became phase dim, not dark like all others except wild-type spores (data not shown), as the spore cortex is not hydrolyzed in spores lacking both CwlJ and SleB.

As noted above, spores of a *B. subtilis* strain lacking multiple YetF family homologs, including both YetF and YrbG, the two most abundant IM homologs (26), spontaneously germinated rapidly on sporulation plates. This was also the case for spores lacking only *yetF* and *yrbG*, as seen in experiments in which spontaneous CaDPA release by sporulating cultures was followed (Fig. 4), and with quadruple mutant spores as measured by microscopy (Fig. S1). In contrast to the rapid spontaneous germination of spores lacking both YetF and YrbG early after their formation noted above, spores lacking YetF alone, YrbG alone, or lacking one, two, or three of the other homologs did not exhibit significant spontaneous germination (data not shown).

The spontaneous germination of spores lacking YetF and YrbG was complete, as the spores became dark in phase contrast microscopy, indicating that both CaDPA release and cortex hydrolysis had taken place (data not shown). An obvious question is why this spontaneous germination takes place. One possibility is that it is because the GRs or the CLEs CwlJ and/or SleB are activated in spores lacking YrbG and YetF, with this activation then triggering germination. Alternatively, perhaps the IM SpoVA proteins’ CaDPA channel opens spontaneously when YetF and YrbG are absent, with the released CaDPA then triggering cortex lysis and completion of germination. To decide between these explanations, we used strains lacking both YetF and YrbG, spores of which germinated rapidly after release from the sporangium (Fig. 4). One additional strain, PS4530, also lacked all five *B. subtilis* GRs, whereas others lacked genes for either of the spores’ two redundant CLEs CwlJ, SleB, or both. Note that spores of all these strains but one (PS4538 lacking both CLEs CwlJ and SleB) have the capacity to release CaDPA and then hydrolyze the spore cortex. However, although *cwlJ sleB* spores can release CaDPA upon GR stimulation, they cannot hydrolyze the spore cortex PG. Consequently, these spores do not complete germination, remaining somewhat bright in phase contrast microscopy, albeit not as bright as spores that do not release CaDPA (27). We then measured CaDPA remaining in wild-type spores and spores of the mutant strains lacking *yetF* and *yrbG* for up to 6 days after inoculation of sporulation plates (Fig. 4). This analysis showed that CaDPA release from *yetF yrbG* spores lacking either all GRs, or either CwlJ or SleB was similar to that from spores lacking *yetF* and *yrbG*. However, CaDPA release by sporulating cells of the *yetF yrbG* strain also lacking *cwlJ* and *sleB* was slower than others except wt spores. Indeed, loss of both CwlJ and SleB has been found to slow CaDPA release in the germination of spores that are otherwise wt (28). In addition, the small amounts of CaDPA retained in spores with the *yrbG yetF* mutations alone or with or without GRs, or CwlJ or SleB, were equal to the small percentages of these spores that were still phase bright 6 days after release from the sporangium (data not shown).

**Fig 5:**
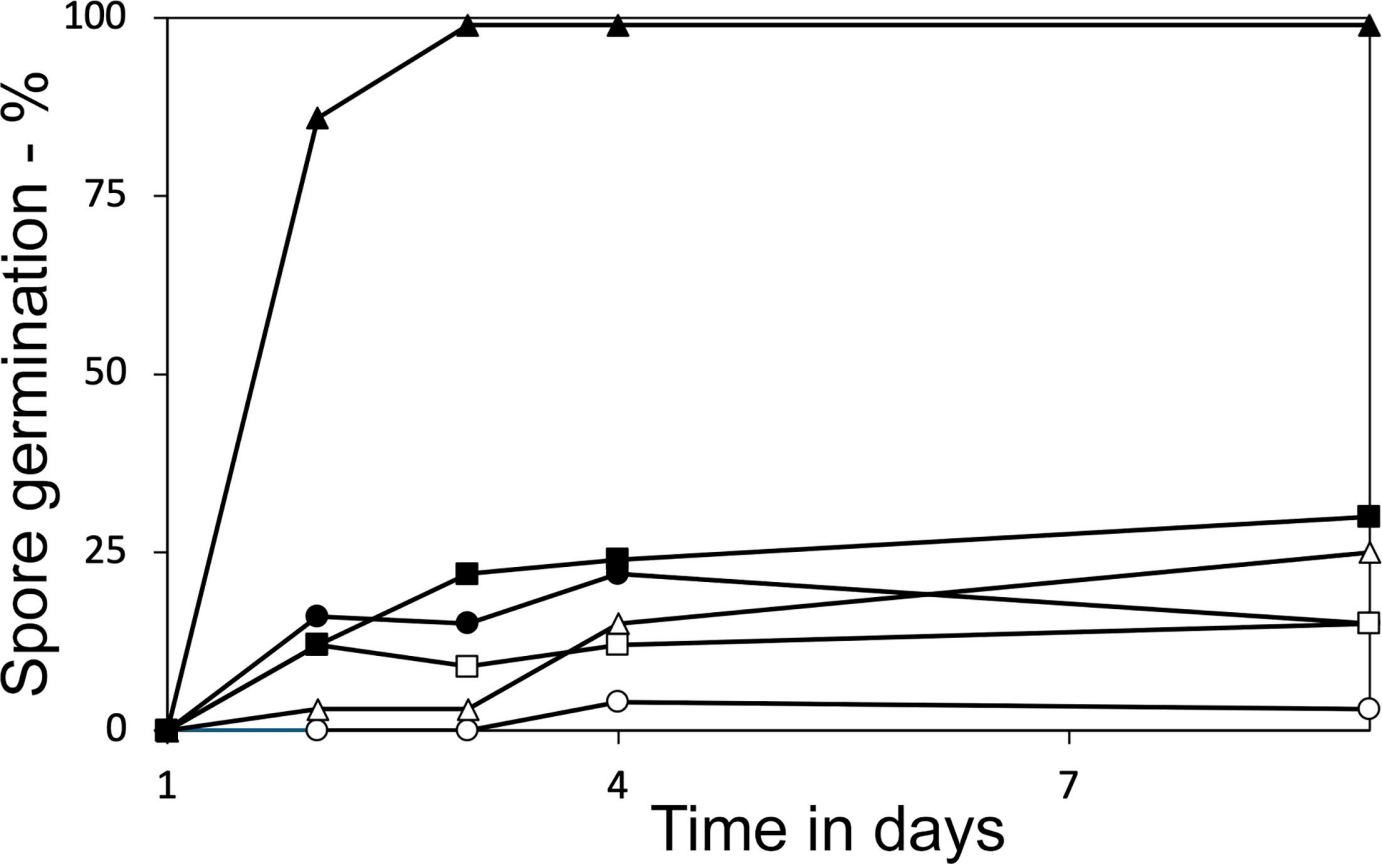
Spontaneous germination of *B. subtilis* spores of various strains with and without 4-fold higher SpoVA protein levels. Growing cells of various strains were spread on sporulation plates, and after 1–9 days, aliquots from plates were examined by phase contrast microscopy, and the percentages of dark (germinated) spores were determined by counting ~150 spores. The strains used and their symbols were: 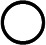, PS832 (wt); 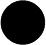, PS3411 (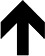
*spoVA*); 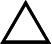
– PS4517 (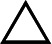
*yrbG*); 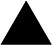, PS4595 (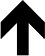
*spoVA* 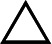
*yrbG*); 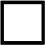, PS4484 (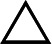
*yetF*); 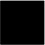, and PS4600 (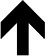
*spoVA yetF*).

Together, the results noted above are consistent with spontaneous opening of the SpoVA channel in the Δ*yrbG* Δ*yetF* spores, causing them to germinate spontaneously, as CaDPA release alone triggers subsequent events in *B. subtilis* spore germination, even in the absence of GRs (27). To test this idea, the promoter strength of the *spoVA* operon was increased ~4-fold (29) in both the wt strain and in strains lacking *yrbG* or *yetF*; these strains were then sporulated on plates, and their germination was measured as well as that of spores of the wt, *yetF,* and *yrbG* strains with the weaker wt *spoVA* promoter (Fig. 5). The results of this experiment were striking, as although the stronger *spoVA* promoter increased spontaneous germination of wt and *yetF* spores to at most ~25% within 7 days, the *yrbG* spores with the stronger *spoVA* promoter spontaneously germinated almost completely in just ~2 days.

### Influence of YetF-type proteins in *B. megaterium* spores

Having established that YetF-type integral IM proteins significantly influence the properties of *B. subtilis* spores, we next sought to determine whether similar observations extended to spores of other Bacillota. The genome of *B. megaterium* QM B1551—a species quite distinct phylogenetically from *B. subtilis*—encodes nine homologs of YetF-type proteins, including one on each of the large indigenous plasmids (Table S3). We decided to attempt to create null deletions in either of the homologs closest in sequence identity to *B. subtilis* YetF (BMQ_2888) or YdfS (BMQ_1954), succeeding with the latter. The resultant *B. megaterium ydfS* strain was found to have spontaneously excised the BM700 plasmid during the mutagenesis procedure, thereby losing the homolog encoded at the BMQ_pBM70026 locus and inadvertently creating a double mutant strain. Spores of the double mutant strain, termed *B. megaterium ydfS* for simplicity, were prepared by nutrient exhaustion in SNB, and sporulation was observed to proceed normally. The spores were then assayed for wet heat resistance and germination properties. Notably, *B. megaterium ydfS* spores are less heat resistant than wt spores, with a near one-log reduction in viability after 30 min at 75°C relative to wt spores and retaining only 5% viability after 1 h of incubation (Fig. 1B). This defect is largely restored by complementing with plasmid-borne *ydfS-gfp*, indicating that the latter fusion is functional. The introduction of plasmid-borne *gerUV* also unexpectedly enhances heat resistance in *ydfS* spores, although viability is still less than half that of wt spores after 30 min at 75°C. The latter observation was unexpected since *B. megaterium* spores that lack the pBM700 plasmid (including g*erUV*) show comparable viability—defined by colony-forming ability on LB plates—to wt spores; hence, perhaps the modest overexpression of *gerUV* from pHT315 has a role in restoring a degree of heat resistance to *ydfS* spores. Differences in heat resistance between wt and *ydfS* spores incubated at 85°C were even more marked, as the viability of *ydfS* spores decreased by 3 logs after 30 min, whereas wt spore viability declined by only 0.5 logs over the same period (Fig. 1B). However, *B. megaterium ydfS* spores with plasmid-borne *gerUV* do not appear to be defective in germination, with their absorbance loss and DPA release displaying similar kinetics to wt spores in response to glucose (Fig. S2). Finally, qualitative analysis by phase contrast microscopy indicates that *B. megaterium ydfS* spores are more prone to spontaneous germination than their wild-type counterparts, regardless of whether plasmid-borne *gerUV* is present (Fig. S3), complementing observations in *B. subtilis* spores that YetF-type proteins contribute to spore stability.

### Location of YetF in spores and relative to the locations of GRs in the germinosome

**Fig 6:**
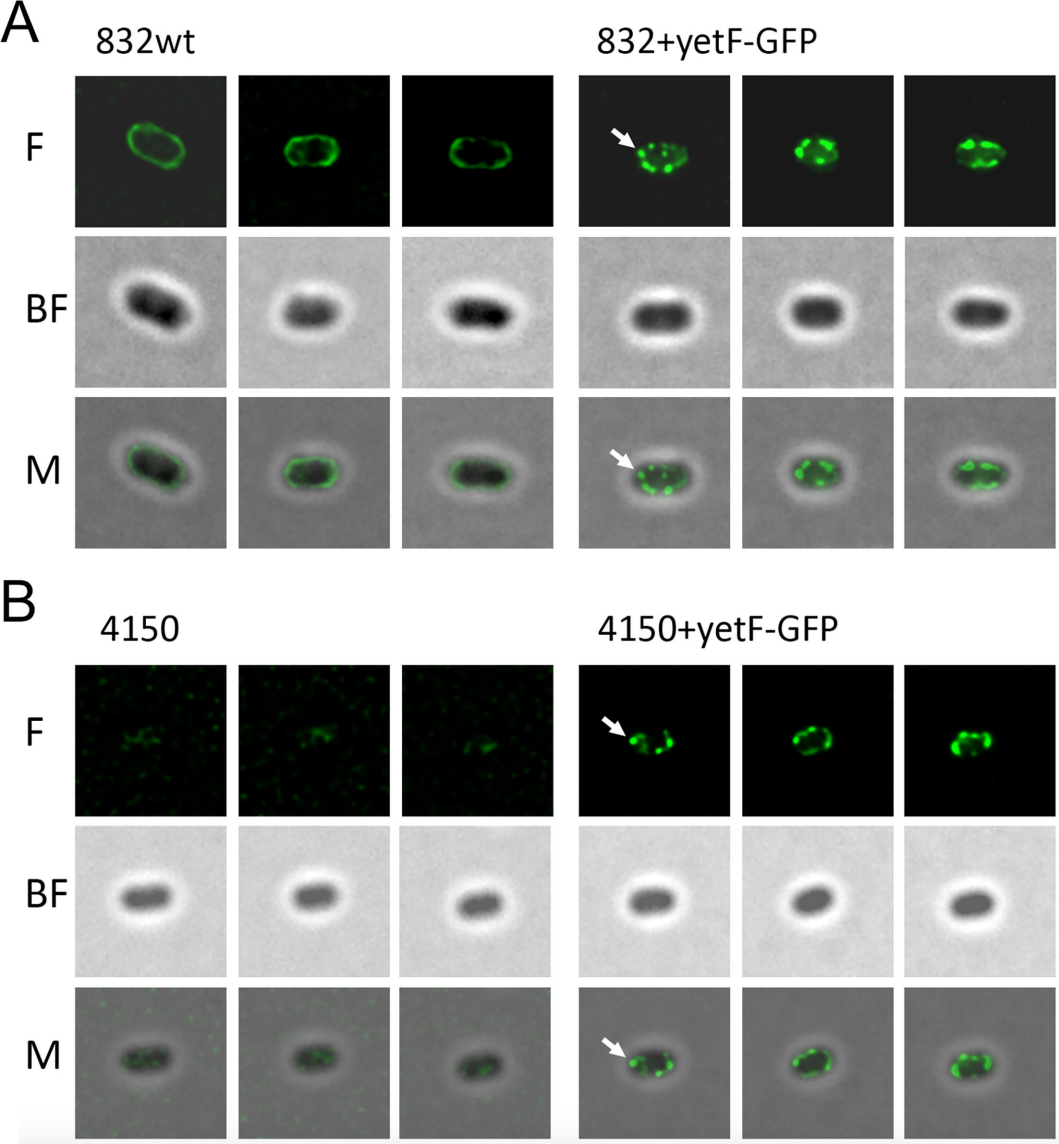
Fluorescence (top images), brightfield (middle images), and merged (bottom panels) images of (A) three wt *B. subtilis* PS832 spores and three PS832 spores with YetF-GFP (PS4603), and (B) three coatless *B. subtilis* PS4150 spores and three PS4150 spores with YetF-GFP (PS4564) obtained as described in Methods. Arrows indicate YetF-GFP locations. Note that the autofluorescence of the spore coat seen in the fluorescence images in (A) is largely absent in the fluorescence images of PS4150 spores in (B).

It is well established that GRs in spores of at least several *Bacillus* species are present in a large complex in spores’ IM termed the germinosome, which is scaffolded by the IM GerD protein (10, 30). Notably, rates of GR-dependent spore germination are increased >10-fold by germinosome formation, and there are generally only 1–2 or 2–3 germinosomes in *B. subtilis* and the larger *Bacillus cereus* spores, respectively (10, 30). Given the effects of the absence of YetF homologs on spore germination as shown above, we made *B. subtilis* YetF-GFP and *B. megaterium* YdfS-GFP fusions and showed that the fusion proteins complemented the effects of a *yetF* or *ydfS* deletion on wet heat resistance of spores of both species and thus are functional (Fig. S4; Fig. 1B). We then examined *B. subtilis* spores with the fusion protein in either strain PS4150 (PS4564) or the wt strain PS832 (PS4603) to determine YetF-GFP locations in spores (Fig. 6A and B). A number of conclusions can be drawn from these images as follows. First, comparison of the bright field and fluorescence images for *B. subtilis* spores with YetF-GFP indicated that this protein is located under the outermost spore layer, consistent with YetF-GFP in the spore IM, where YetF has been located by proteomic studies (26) and western blot analysis of YetF-GFP in soluble and membrane fractions of spores expressing this protein (data not shown). Note that a likely IM location of YetF-GFP was also found previously in sporulating cells of *B. subtilis* (31). Second, the YetF-GFP is clearly not uniformly distributed in the IM of wt or fully coatless *B. subtilis* spores, as most of these spores show 5–7 separate spots of green fluorescence in the IM, many more than the one or less often two germinosomes present in *B. subtilis* spores (10). Third, the YetF-GFP spots are spread around the spore, again not consistent with YetF-GFP alignment with the germinosome. One possibility, however, is that YetF-GFP, and presumably YetF itself, is located in or near the multiple places in the IM where late in sporulation the IM is pushed into the spore core (32). If so, examination of the YetF-GFP location in a germinated spore might be illuminating.

*B. megaterium* wt spores carrying YetF-GFP displayed a similar distribution of fluorescent foci as observed in the corresponding *B. subtilis* strain (Fig. S5). In contrast*, B. megaterium* YdfS-GFP is present in only a single fluorescent focus, as observed in wt, *ydfS,* and the otherwise isogenic PV361 strains carrying the *ydfS-gfp* construct (the latter strain lacks all seven indigenous plasmids) (Fig. S5 and data not shown). Attempts to ascertain whether the YdfS focus co-localizes with GerUA-GFP, and therefore, the *B. megaterium* germinosome were indeterminate, although most spores were shown to have 1–3 germinosomes (data not shown).

### IM properties in spores lacking YetF homologs

YetF homologs are IM proteins, and their loss has dramatic effects on two spore properties, resistance and germination, in which IM properties play major roles (2, 33). Consequently, it was important to look at the effects of loss of these homologs on spore IM permeability and fluidity/rigidity. These studies found that loss of three homologs in strain PS4531 unexpectedly slowed the rate of passage of methylamine into the spore core compared with that in wild-type spores, whereas the loss of YetF alone caused less of a decrease in methylamine uptake (Fig. S6). Note also that the rate of methylamine uptake by spores of the coatless strain PS4150 was faster than in wt spores (Fig. S6), consistent with the significantly increased IM permeability to water in the coatless spores (34). However, in contrast to the methylamine permeability results, FRAP analysis showed that PS4531 mutant spores’ IM fluidity had increased significantly, as the diffusion coefficient of a dye in spores’ IM was significantly higher in 4531 spores than in wt spores (Table S2). This finding was supported by analysis of IM fluidity by measuring the fluorescence emission of the dye Laurdan in spores’ IM, as the fluorescence emission spectra change differently in more fluid than in more rigid membrane environments, as measured by its GP value (Table S2), which decreases in a more fluid IM environment (6, 24). Note that the extremely fluid IM values in germinated spores, as measured using the Laurdan dye (Table S2), are consistent with conclusions based on FRAP analyses carried out some years ago (20). Notably, the change in Laurdan fluorescence in germinated spores compared with wt spores was so great that it was easily seen by eye in fluorescence micrographs (Fig. S7).

## DISCUSSION

The new results from this work have significant implications for a thorough understanding of spore resistance and germination in a number of ways. First, it is clear, as suggested previously for two YetF homologs, that all of these integral IM proteins play a significant role in spore resistance to wet heat, H_2_O_2_, and HCHO. Presented data indicate that this unspecified role in heat resistance, at least, can be extended to *B. megaterium* and probably all Bacillota spores. Since H_2_O_2_ and HCHO kill spores by damaging either DNA (HCHO) or a core protein(s) (H_2_O_2_), these agents must cross the IM, where YetF homologs alter IM properties (33). In addition, wet heat has been proposed to kill spores by damaging IM enzymes essential for ATP generation, and spore wet heat resistance has been suggested to be largely due to IM protein protection from wet heat by their embedding in a relatively rigid IM (5, 23). These observations suggest that the effects of YetF homologs on IM permeability, fluidity, and rigidity may be important in understanding the effects of these homologs on spore resistance and germination. FRAP analyses, as well as Laurdan fluorescence using wt spores and mutant spores lacking three YetF homologs, both with the reporter dyes in the IM, indicated that the IM in mutant spores was more fluid than in wt spores. This observation seems at odds with the similar methylamine permeability in wt and mutant spores, but perhaps methylamine permeability is not directly linked to IM fluidity, and formaldehyde and hydrogen peroxide seem likely to exhibit lower permeability than uncharged methylamine.

The second notable observation from the current work is the spontaneous germination soon after spores missing YetF homologs are released from the mother cell. Although such spontaneous germination has been seen previously (35), this was by activation of the GerA GR in sporulating *B. subtilis*. In contrast, the spontaneous germination of spores lacking YetF and YrbG seen in the current work was independent of both GRs and CLEs and is most likely due to spontaneous opening of the IM SpoVA channel, in particular, the SpoVAC mechanosensitive IM protein through which CaDPA is most likely released (36). Why this channel should open is not completely clear, although it is certainly possible that if the fluidity of the IM in a dormant spore increases sufficiently, then this might trigger spontaneous SpoVAC opening through random protein motions.

The third notable observation was that the YetF homologs affect spore germination, with some increasing germination and others decreasing it. Certainly, effects on IM fluidity could play roles here, and there is previous work showing that decreases in IM fluidity are associated with slower GR-dependent germination (23). However, why different YetF homologs have different effects on germination is not at all clear. One possibility is that YetF or YrbG is associated with the germinosome, or essential for germinosome assembly in some fashion. However, this seems unlikely for at least YetF, as YetF-GFP was in 5–7 spots, many more than the 1–2 germinosomes in *B. subtilis* spores (10); perhaps, YrbG takes up different positions in spores than does YetF? This seems to be the case for *B. megaterium* YdfS and YetF, the former localizing to single spots per spore and the latter multiple. In common with spores of other species, *B. megaterium* has typically 1–3 germinosomes per spore, and it is not inconceivable that the loss of heat resistance observed in *ydfS* null spores reflects a stabilizing role for YdfS within these assemblies of germinant receptor proteins. Future work in this area will seek to address this possibility and other functional aspects associated with YetF-type proteins in spore resistance and germination.

## SUPPLEMENTAL MATERIAL

Supplemental tables and figures jb.00217-25-s0001.pdf Tables S1 to S3 and Fig. S1 to S7.

ASM does not own the copyrights to Supplemental Material that may be linked to, or accessed through, an article. The authors have granted ASM a non-exclusive, world-wide license to publish the Supplemental Material files. Please contact the corresponding author directly for reuse.
