# Supplementary material for: Resistance and germination of spores of Bacillus species lacking members of a spore integral inner membrane protein family and locations of these proteins in spores
Source: J Bacteriol. 2025 Sep 12;207(10):e00217-25. doi: 10.1128/jb.00217-25 (PMC12548441; doi:10.1128/jb.00217-25)
Supplement: Supplemental tables and figures — Tables S1 to S3 and Fig. S1 to S7. [file jb.00217-25-s0001.pdf]

Supplemental Information

Resistance and Germination of Spores of *Bacillus* Species Lacking Members of a Spore Integral Inner Membrane Protein Family and Locations of These Proteins in Spores

Shermeen Khan, James Wicander, George Korza, Rebecca Caldbeck, Ann E. Cowan, Graham Christie and Peter Setlow

This file includes:

Supplemental Tables 1 - 3

Supplemental Figures 1 – 7

Table S1 Bacterial strains used in this work*

Strain identifier	Deleted/altered genes	Antibiotic resistance	Reference
Bacillus subtilis strains			
PS832	none	none	lab strain
PS533	Carries plasmid pUB110	Km^r	(1)
PS3411	↑*spoVA* operon	Em^r	(2)
PS3518	P*sspE-gfp*	Km^r	(3)
PS4150	*cotE gerE*	Sp^r Tet^r	(4)
PS4483	*ydfS*	Em^r	(5)
PS4484	*yetF*	Em^r	(5)
PS4501	*gerA gerB gerK yndD yfkQ*	none	this work
PS4517	*yrbG*	Km^r	this work
PS4518	*ykjA*	Km^r	this work
PS4519	*ydfR*	Em^r	this work
PS4520	*yrbG ydfR*	Em^r Km^r	this work
PS4521	*yrbG ydfR*	none	this work
PS4524	*yrbG ydfR ykjA*	Km^r	this work
PS4525	*yrbG ykjA yetF*	Km^r Em^r	this work
PS4526	*yrbG ykjA yetF*	none	this work
PS4530	*yrbG ykjA yetF ydfS*	none	this work
PS4531	*yrbG ykjA ydfS*	Km^r Em^r	this work
PS4532	*yetF yrbG ydfS ykjA gerAA*	Em^r Sp^r	this work
PS4533	*yrbG yetF*	Km^r Em^r	this work
PS4534	*ydfS ykjA*	Km^r Em^r	this work
PS4535	*ydfS ydjA*	none	this work
PS4536	*yetF yrbG ydfS ykjA cwlJ*	Em^r Tc^r	this work
PS4537	*yetF yrbG ydfS ykjA sleB*	Em^r Km^r Sp^r	this work
PS4538	*yetF yrbG ydfS ykjA cwlJ sleB*	Em^r Km^r Sp^r Tc^r	this work
PS4541	Δ*yrbG* Δ*yetF* in PS4501	Em^r Km^r	this work
PS4563	*gerKB-mCherry yetF-gfp*	Em^r Km^r Sp^r Tc^r Cm^r	this work
PS4564[1]	*yetF-gfp* in PS4150	Sp^r Tc^r Cm^r	this work
PS4565[1]	P*sspE-gfp*	Sp^r Tc^r Km^r	(3)
PS4566[1]	*yetF-gfp* Δ*yetF*	Sp^r Tc^r Cm^r Em^r	this work
PS4567[1]	*gerKB*::*gerKB-gfp* Δ*yetF*	Em^r Km^r Sp^r Tc^r	this work
PS4568[1]	*gerKB*::*gerKB-gfp* Δ*yetG*	Em^r Km^r Sp^r Tc^r	this work
PS4577	4484 without antibiotic marker	none	This work

PS4595	↑*spoVA* operon Δ*yrbG*	Em^r Km^r	this work
PS4600	↑*spoVA* operon Δ*yetF*	Em^r Km^r	this work
PS4601[2]	*yrbG ydfS ykjA*	none	this work
PS4602[2]	*yrbG ydfR ykjA*	none	this work
PS4603	*yetF-gfp* in PS832	Cm^r	this work
FB20	*gerAA*	Sp^r	(6)
FB111	*cwlJ*	Tc^r	(7)
FB113	*cwlJ sleB*	Sp^r Tc^r	(7)
KGB199[1]	*gerKB-mCherry*	Km^r Em^r	(8)
Bacillus megaterium strains			
QM B1551	Wild type	none	Pat Vary
RC010[3]	*ydfS* (*BMQ_1954*)	Km^r	This work
RC011[3]	*pHT-ydfS-gfp* in RC010	Km^r Em^r	This work
RC012[3]	*pHT-gerUV* in RC010	Km^r Em^r	This work
RC013	*pHT-yetF-gfp*	Em^r	This work
RC014[4]	*pHT-gerUV-gfp*	Em^r	This work

*Strains or plasmids made in this work were constructed as described in Methods, and antibiotic resistances are to: erythromycin (Em^r), 5 μg/ml; kanamycin (Km^r), 10 μg/ml; spectinomycin (Sp^r), 50 μg/ml; tetracycline (Tc^r), 10 μg/ml; and chloramphenicol (Cm^r) (10 μg/ml). All strains except those labelled [1] were constructed in the PS832 background; those labelled [2] were in the PS4150 background.

[2]The antibiotic markers were removed from strains 4524 and PS4531 by the Cre recombinase (9).

[3]These strains lack the pBM700 plasmid which includes the BMQ_pBM70026 YetF homologue.

[4] This strain is designed to express GerUA-GFP as a component of the GerUV germinant receptor.

Table S2 Inner membrane fluidity in spores of various strains as measured by either FRAP analysis using di-4-ANEPPS or changes in Laurdan fluorescence

FRAP analysis[1]		
Spores examined	Average IM diffusion coefficient	Mobile lipid fraction
PS832 (wt)	0.21 +/- 0.12	0.25 +/- 0.15
PS4531 (Δ*yrbG ykjA ydfS*)	0.81 +/- 0.34	0.19 +/- 0.07

Laurdan analysis[2]		
	Laurdan GP[3]	Number of spores examined
Dormant/Decoated PS832 (wt)	0.27 +/- 0.05	30
Dormant/Decoated PS4531 (Δ*yrbG ykjA ydfR*)	0.14 +/- 0.05	30
Decoated/Germinated PS832 (wt)	0.01 +/- 0.02	30

[1]Spore labeling with 2,4-di-ANEPPS and procedures for FRAP analyses are described in Methods, and 9 individual PS832 and 10 individual PS4531 spores were analyzed.

[2]Spores were labeled with Laurdan, purified and decoated and their Laurdan fluorescence intensities from spores at different wavelengths were determined in dormant and germinated spores, all as described in Methods.

[3]$GP = (I_{440}-I_{490})/(I_{440}+I_{490})$

Table S3 *Bacillus megaterium* QM B1551 YetF homologues

Locus	Identity (%)[1]	Positives (%)[2]	Coverage (%)	E-value
BMQ_2888	53	72	93	1e-85
BMQ_0744	51	69	97	2e-84
BMQ_3181	35	54	92	1e-33
BMQ_2721	35	57	95	1e-44
BMQ_pBM70026	33	53	59	9e-16
BMQ_1954	32	56	97	1e-38
BMQ_pBM60079	32	55	89	3e-33
BMQ_1938	31	53	97	4e-34
BMQ_4637	26	49	87	6e-19

[1] Amino acid sequence identity with *B. subtilis* YetF

[2] Positives = sum of identical and similar residues

Fig. S1.

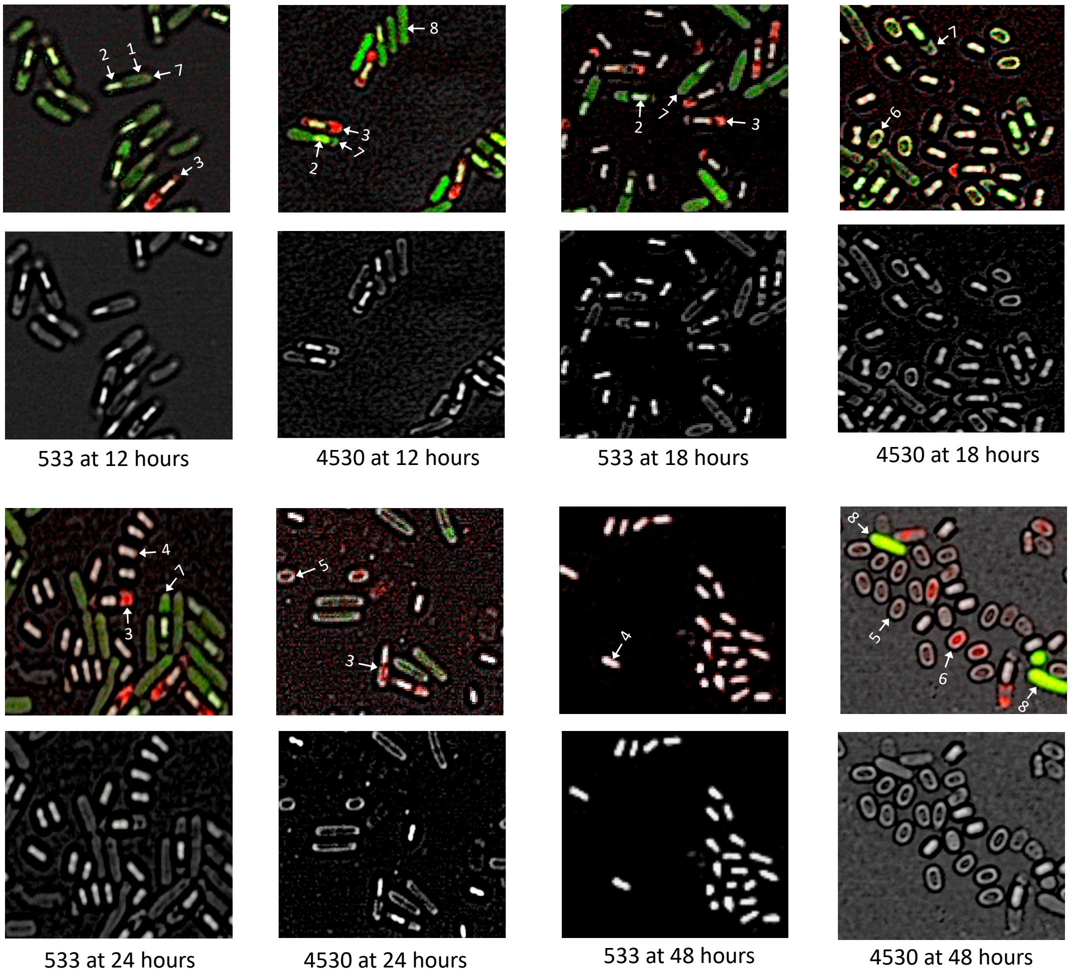

Fig. S1. Fluorescence (upper panels) and brightfield (lower panels) micrographs of sporulating cultures of *B. subtilis* strains PS533 (wild-type) and PS4530 (lacking all YetF homologs but YdfR) undergoing spontaneous germination. Samples were stained with the BacLight reagent after harvest of samples into cold water at various times after cells were spread on sporulation plates, and fluorescence and brightfield images were collected as described in Methods. The arrows designate: 1, sporangium with a spore; 2, bright spores in a sporangium; 3, likely dead mother cell compartments staining red; 4, free bright spores; 5, free dim spores (no stain uptake); 6, free dim spores (stained); 7, likely live mother cell compartments staining green; 8, live cell that hasn't initiated sporulation. The arrow labeled 6 for strain PS4530 at 18 hr is an example of a green dim spore which is likely still alive,

whereas arrow 6 for strain PS4530 at 48 hr is an example of a dim red spore that is likely dead.

Fig. S2.

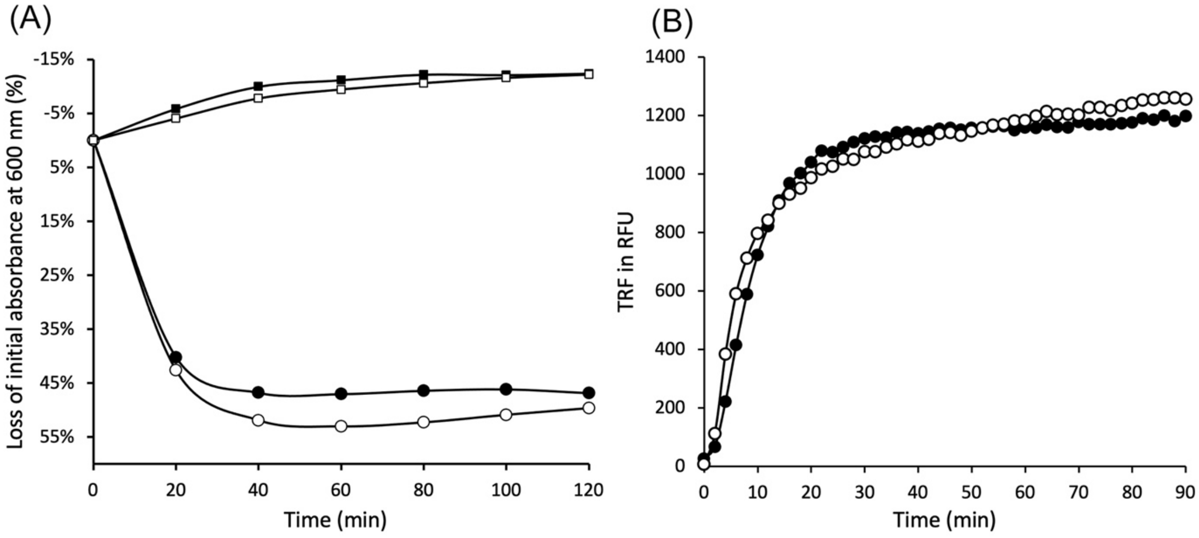

Fig. S2. Germination of *Bacillus megaterium* in 5 mM Tris-HCl, pH 7.5, supplemented with 10 mM glucose. Spores were heat shocked (60°C for 10 min) and cooled before resuspending in buffer and germination monitored by (A) absorbance at 600 nm, and (B) CaDPA release by $TbCl_3$ fluorescence assay. Presented data are from single experiments, which are representative of analyses conducted with at least two independent batches of spores and where SD from mean values is <10%. Key: Wild type with (○) and without (□) glucose; Δ*ydfS* with (●) and without (■) glucose.

Fig. S3.

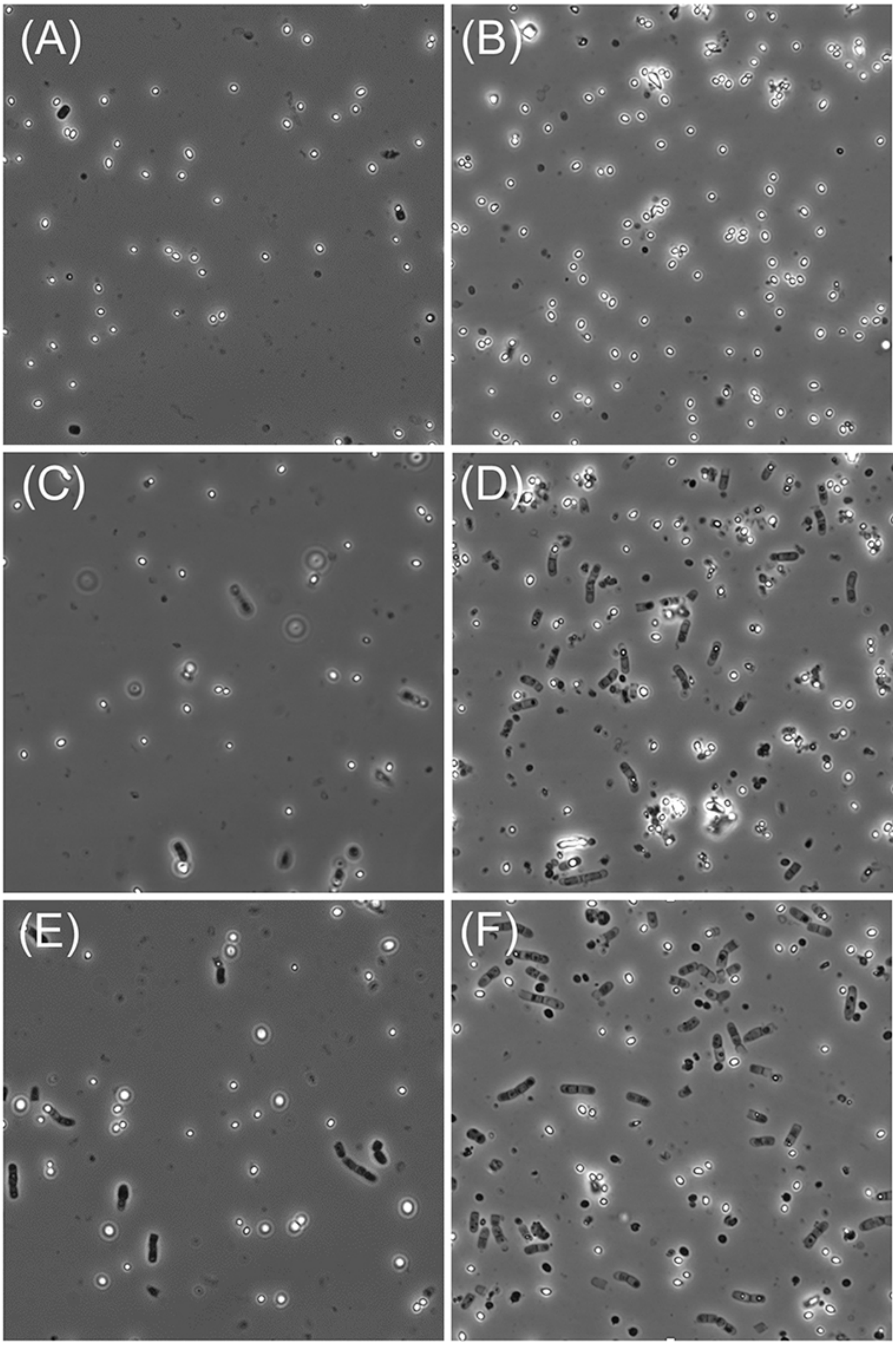

Fig. S3. Phase contrast microscopy images of *Bacillus megaterium* cultures after 2 days and 25 days in supplemented nutrient broth. Cells were cultured at 30°C in shake flasks (100 rpm) for 2 days and imaged, and then maintained at 25°C, 100 rpm, until day 25 and imaged again.

Key: (A) and (B), wild type at 2 d and 25 d respectively; (C) and (D), Δ*ydfS* at 2 d and 25 d respectively; (E) and (F), Δ*ydfS* with plasmid borne *gerUV* at 2 d and 25 d respectively.

Fig. S4.

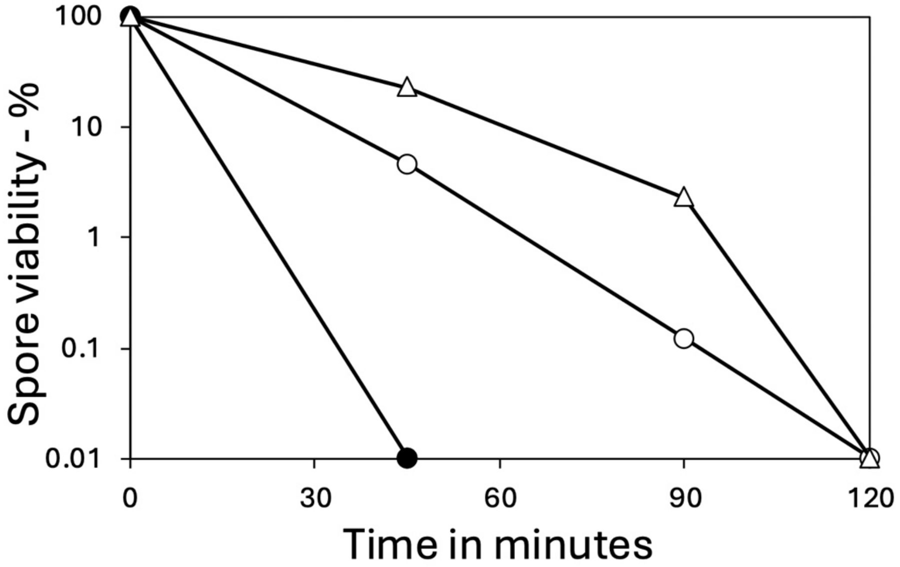

Fig. S4. Complementation of *B. subtilis yetF* mutant spores' decreased wet heat resistance by YetF-GFP. The measurement of wet heat killing of spores of *B. subtilis* strains was carried out at 93°C in duplicate in one experiment as described in Methods; the symbols used are: (○) PS832 (wt), (●) PS4484 (Δ*yetF*) and (△) PS4566 (Δ*yetF yetF-gfp*).

Fig. S5.

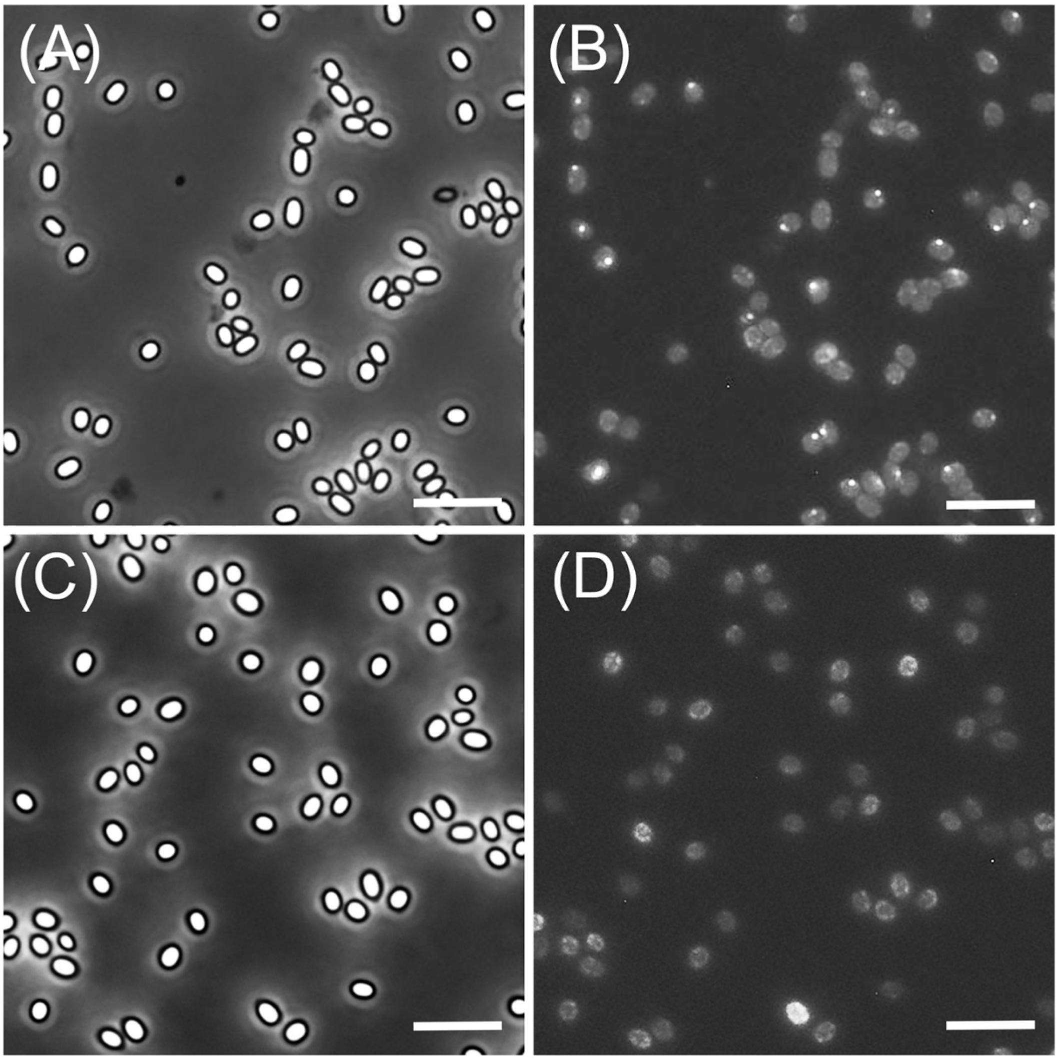

Fig. S5. Phase contrast and fluorescence microscopy images of *B. megaterium ydfS-gfp* and *yetF-gfp* spores. Key: *B. megaterium ydfS-gfp* (A) phase and (B) fluorescence images; *B. megaterium yetF-gfp* (C) phase and (D) fluorescence images. YdfS-GFP appears to predominantly cluster in single fluorescent foci in individual spores whereas YetF-GFP forms multiple foci. Scale bar represents 5 μm.

Fig. S6.

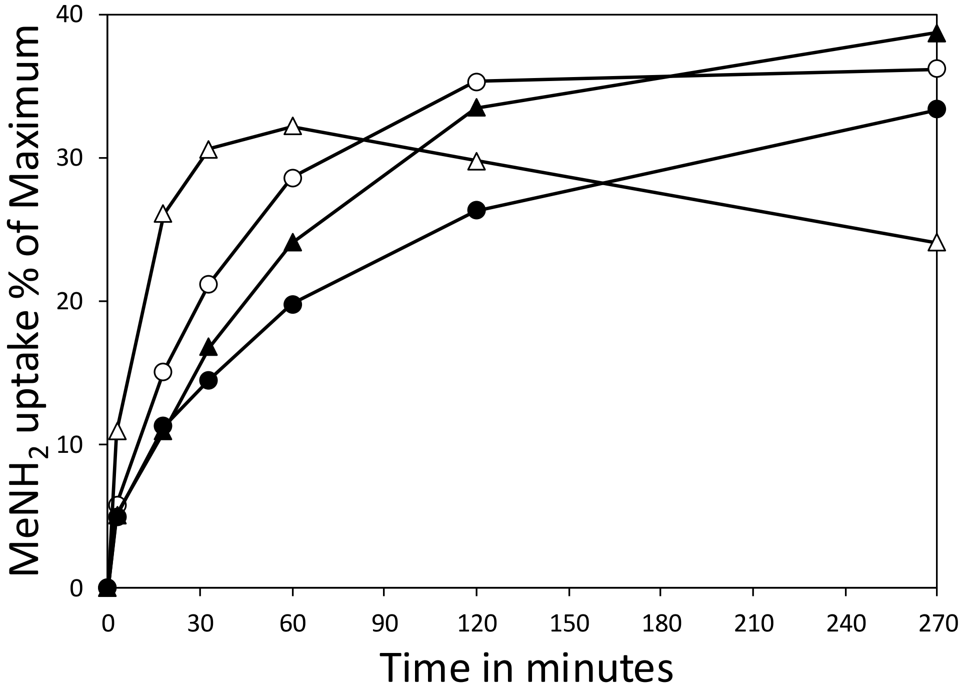

Fig. S6. Methylamine uptake by wt and mutant spores. Methylamine uptake by spores of strains PS832 (wt, ○); PS4150 (Δ*cotE* Δ*gerE*, △); PS4531 (Δ*yrbG* Δ*ykjA* Δ*ydfR*, ●) and 4577 (Δ*yetF*, ▲ - no antibiotic resistance), was measured as described in Methods. The maximum value was the total amount of ^{14}C-methylamine in samples taken for analysis of methylamine uptake - ~177,000 cpm.

Fig. S7.

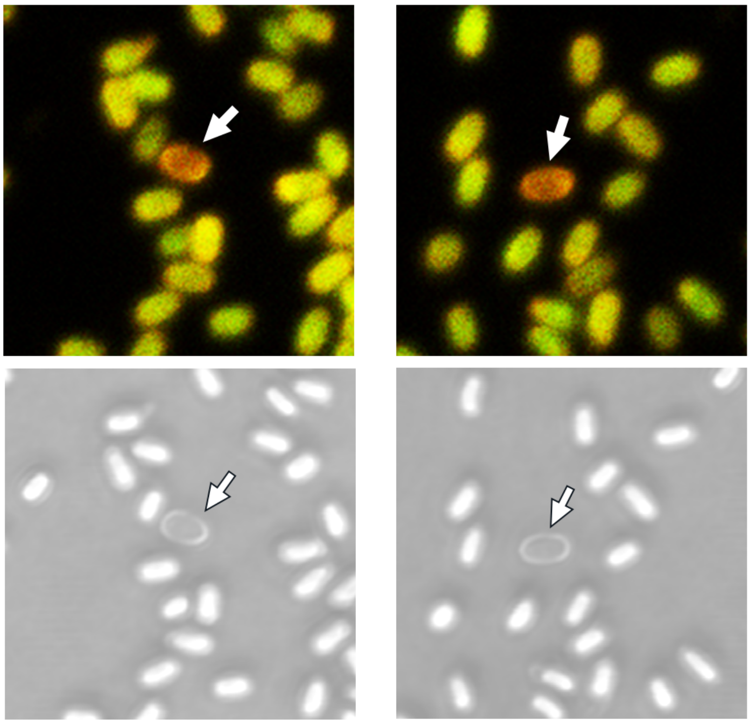

Fig. S7. Fluorescent (upper panels) and brightfield (lower panels) images of wt *B. subtilis* PS832 spores stained with Laurdan as described in Methods. The arrows designate germinated spores.

References

1. Setlow B, Setlow P. 1996. Role of DNA repair in *Bacillus subtilis* spore resistance. J Bacteriol 178(12):3486-95.

2. Vepachedu VR, Setlow P. 2007. Role of SpoVA proteins in release of dipicolinic acid during germination of *Bacillus subtilis* spores triggered by dodecylamine or lysozyme. J Bacteriol 189(5):1565-72.

3. Cowan AE, Koppel DE, Setlow B, Setlow P. 2003. A soluble protein is immobile in dormant spores of *Bacillus subtilis* but is mobile in germinated spores: implications for spore dormancy. Proc Natl Acad Sci U S A 100(7):4209-14.

4. Ghosh S, Setlow B, Wahome PG, Cowan AE, Plomp M, Malkin AJ, Setlow P. 2008. Characterization of spores of *Bacillus subtilis* that lack most coat layers. J Bacteriol 190(20):6741-8.

5. Yu B, Kanaan J, Shames H, Wicander J, Aryal M, Li Y, Korza G, Brul S, Kramer G, Li YQ, Nichols FC, Hao B, Setlow P. 2023. Identification and characterization of new proteins crucial for bacterial spore resistance and germination. Front Microbiol 14:1161604.

6. Paidhungat M, Setlow P. 2000. Role of ger proteins in nutrient and nonnutrient triggering of spore germination in *Bacillus subtilis*. J Bacteriol 182(9):2513-9.

7. Paidhungat M, Ragkousi K, Setlow P. 2001. Genetic requirements for induction of germination of spores of *Bacillus subtilis* by Ca(2+)-dipicolinate. J Bacteriol 183(16):4886-93.

8. Griffiths KK, Zhang J, Cowan AE, Yu J, Setlow P. 2011. Germination proteins in the inner membrane of dormant *Bacillus subtilis* spores colocalize in a discrete cluster. Mol Microbiol 81(4):1061-77.

9. Koo BM, Kritikos G, Farelli JD, Todor H, Tong K, Kimsey H, Wapinski I, Galardini M, Cabal A, Peters JM, Hachmann AB, Rudner DZ, Allen KN, Typas A, Gross CA. 2017. Construction and analysis of two genome-scale deletion libraries for *Bacillus subtilis*. Cell Syst 4(3):291-305 e7.
